# (*E*)-3-Hy­droxy-5,5-dimethyl-2-(3-phenyl­prop-2-en-1-yl)cyclo­hex-2-en-1-one

**DOI:** 10.1107/S160053681103220X

**Published:** 2011-08-17

**Authors:** Afsaneh Zonouzi, Zakieh Izakiana, Seik Weng Ng

**Affiliations:** a Department of Chemistry, College of Science, University of Tehran, PO Box 14155-6455 Tehran, Iran; b Department of Chemistry, University of Malaya, 50603 Kuala Lumpur, Malaysia; c Chemistry Department, Faculty of Science, King Abdulaziz University, PO Box 80203 Jeddah, Saudi Arabia

## Abstract

Five of the atoms of the six-membered cyclo­hexene ring of the title compound, C_17_H_20_O_2_, are essentially coplanar (r.m.s. deviation = 0.006 Å), with the sixth (the dimethyl­methyl C atom) deviating from the mean plane of the five atoms by 0.610 (2) Å. This plane is nearly perpendicular to the cinnamyl portion, the two planes being aligned at 85.1 (1)°. Two mol­ecules are linked by an O—H⋯O hydrogen bond about a center of inversion. The cyclo­hexene ring is disordered over two directly overlapping positions. As a result, the hy­droxy group and the keto O atom cannot be distinguished from one another.

## Related literature

For the synthesis, see: Gan *et al.* (2008 ▶).
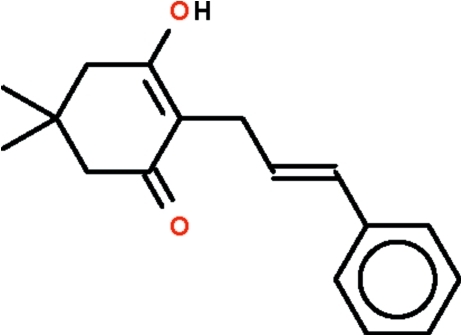


## Experimental

### 

#### Crystal data

C_17_H_20_O_2_
*M* *_r_* = 256.33
Triclinic, 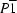

*a* = 5.6480 (2) Å
*b* = 10.9077 (5) Å
*c* = 12.4762 (8) Å
α = 70.999 (5)°
β = 89.533 (4)°
γ = 75.783 (4)°
*V* = 702.31 (6) Å^3^
*Z* = 2
Mo *K*α radiation
μ = 0.08 mm^−1^
*T* = 100 K
0.30 × 0.25 × 0.20 mm

#### Data collection

Agilent SuperNova Dual diffractometer with an Atlas detector
Absorption correction: multi-scan (*CrysAlis PRO*; Agilent, 2010 ▶) *T*_min_ = 0.977, *T*_max_ = 0.985
6734 measured reflections
3119 independent reflections
2554 reflections with *I* > 2σ(*I*)
*R*_int_ = 0.026

#### Refinement

*R*[*F* ^2^ > 2σ(*F* ^2^)] = 0.045
*wR*(*F* ^2^) = 0.120
*S* = 1.02
3119 reflections
174 parameters
H-atom parameters constrained
Δρ_max_ = 0.29 e Å^−3^
Δρ_min_ = −0.21 e Å^−3^

### 

Data collection: *CrysAlis PRO* (Agilent, 2010 ▶); cell refinement: *CrysAlis PRO*; data reduction: *CrysAlis PRO*; program(s) used to solve structure: *SHELXS97* (Sheldrick, 2008 ▶); program(s) used to refine structure: *SHELXL97* (Sheldrick, 2008 ▶); molecular graphics: *X-SEED* (Barbour, 2001 ▶); software used to prepare material for publication: *publCIF* (Westrip, 2010 ▶).

## Supplementary Material

Crystal structure: contains datablock(s) global, I. DOI: 10.1107/S160053681103220X/bt5605sup1.cif

Structure factors: contains datablock(s) I. DOI: 10.1107/S160053681103220X/bt5605Isup2.hkl

Supplementary material file. DOI: 10.1107/S160053681103220X/bt5605Isup3.cml

Additional supplementary materials:  crystallographic information; 3D view; checkCIF report

## Figures and Tables

**Table 1:** Hydrogen-bond geometry (Å, °)

*D*—H⋯*A*	*D*—H	H⋯*A*	*D*⋯*A*	*D*—H⋯*A*
O1—H1⋯O1^i^	0.84	1.76	2.582 (2)	166
O2—H2⋯O2^ii^	0.84	1.74	2.569 (2)	167

Symmetry codes: (i) 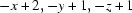; (ii) 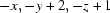.

**Fig. 1.**
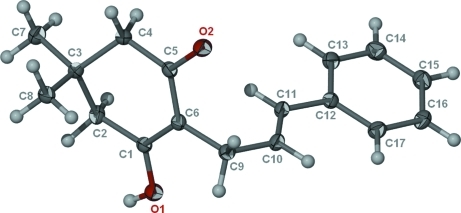
Thermal ellipsoid plot (Barbour, 2001) of C17H20O2 at the 70% probability level; hydrogen atoms are drawn as spheres of arbitrary radius.

## supplementary crystallographic information

### Comment

Dimedone condenses with aromatic aldehydes. Cinnamaldehyde is the aldehyde in the present study. The compound C_17_H_20_O_2_ (Scheme I, Fig. 1) possess a hydroxy as well as a ketonic unit; these interact by an O–H···O hydrogen bond to generate a hydrogen-bonded dimer (Table 1). The synthesis illustrates the direct activation of a C–O bond of a cyclic 1,3-dione to yield the *C*-alkylated product; an earlier report detailed the synthesis that is catalyzed by palladium compounds (Gan *et al.*, 2008). Five of the atoms of the six-membered cyclohexene ring of C_17_H_20_O_2_ are coplanar, with the sixth (the dimethylmethyl carbon) deviating from the mean-plane of the five. This plane is perpendicular to the cinnamyl portion. Two molecules are linked by an O–H···O hydrogen bond about a center-of-inversion (Table 1).

### Experimental

To a stirred solution of dimedone (0.68 g, 5 mmol) and cinnamaldehyde (0.66 g, 5 mmol) in ethanol (10 ml) was added zinc chloride (1 mmol) along with a small amount of a primary amine as catalyst. The mixture was heated for 12 h. The product was purified by column chromatography on silica gel by using an ethyl acetate/*n*-hexane (1:3) solvent system. The compound was recrystallized from ethyl acetate to give colorless crystals (yield 70%).

### Refinement

Carbon-bound H-atoms were placed in calculated positions [C—H 0.95 to 0.98 Å, *U*_iso_(H) 1.2 to 1.5*U*_eq_(C)] and were included in the refinement in the riding model approximation.

The hydroxy H-atom is disordered over two positions, and the occupancy was assumed to be 0.5. The two half-occupancy atoms were already treated as riding [O—H 0.84 Å, *U*_iso_(H) 1.5*U*_eq_(O)].

### Crystal data

**Table:**

C_17_H_20_O_2_	*Z* = 2
*M**_r_* = 256.33	*F*(000) = 276
Triclinic, *P*1	*D*_x_ = 1.212 Mg m^−^^3^
Hall symbol: -P 1	Mo *K*α radiation, λ = 0.71073 Å
*a* = 5.6480 (2) Å	Cell parameters from 3442 reflections
*b* = 10.9077 (5) Å	θ = 2.3–29.3°
*c* = 12.4762 (8) Å	µ = 0.08 mm^−^^1^
α = 70.999 (5)°	*T* = 100 K
β = 89.533 (4)°	Block, colorless
γ = 75.783 (4)°	0.30 × 0.25 × 0.20 mm
*V* = 702.31 (6) Å^3^	

### Data collection

**Table:**

Agilent SuperNova Dual diffractometer with an Atlas detector	3119 independent reflections
Radiation source: SuperNova (Mo) X-ray Source	2554 reflections with *I* > 2σ(*I*)
Mirror	*R*_int_ = 0.026
Detector resolution: 10.4041 pixels mm^-1^	θ_max_ = 27.5°, θ_min_ = 3.1°
ω scans	*h* = −7→7
Absorption correction: multi-scan (*CrysAlis PRO*; Agilent, 2010)	*k* = −14→14
*T*_min_ = 0.977, *T*_max_ = 0.985	*l* = −11→16
6734 measured reflections	

### Refinement

**Table:**

Refinement on *F*^2^	Primary atom site location: structure-invariant direct methods
Least-squares matrix: full	Secondary atom site location: difference Fourier map
*R*[*F*^2^ > 2σ(*F*^2^)] = 0.045	Hydrogen site location: inferred from neighbouring sites
*wR*(*F*^2^) = 0.120	H-atom parameters constrained
*S* = 1.02	*w* = 1/[σ^2^(*F*_o_^2^) + (0.0515*P*)^2^ + 0.1884*P*] where *P* = (*F*_o_^2^ + 2*F*_c_^2^)/3
3119 reflections	(Δ/σ)_max_ = 0.001
174 parameters	Δρ_max_ = 0.29 e Å^−^^3^
0 restraints	Δρ_min_ = −0.21 e Å^−^^3^

### Fractional atomic coordinates and isotropic or equivalent isotropic displacement parameters (Å^2^)

**Table:**

	*x*	*y*	*z*	*U*_iso_*/*U*_eq_	Occ. (<1)
O1	0.86917 (17)	0.62115 (9)	0.45112 (9)	0.0271 (3)	
H1	0.9361	0.5411	0.4900	0.041*	0.50
O2	0.19642 (18)	0.96431 (9)	0.45314 (9)	0.0290 (3)	
H2	0.0794	0.9797	0.4929	0.044*	0.50
C1	0.6812 (2)	0.66673 (13)	0.50012 (11)	0.0197 (3)	
C2	0.6155 (3)	0.57371 (13)	0.60766 (12)	0.0233 (3)	
H2A	0.7686	0.5134	0.6523	0.028*	
H2B	0.5231	0.5172	0.5873	0.028*	
C3	0.4630 (2)	0.64378 (13)	0.68296 (12)	0.0205 (3)	
C4	0.2525 (2)	0.75600 (14)	0.60738 (12)	0.0232 (3)	
H4A	0.1305	0.7150	0.5857	0.028*	
H4B	0.1708	0.8122	0.6521	0.028*	
C5	0.3312 (2)	0.84458 (13)	0.50111 (11)	0.0205 (3)	
C6	0.5413 (2)	0.79848 (13)	0.45053 (11)	0.0204 (3)	
C7	0.3597 (3)	0.54314 (14)	0.77432 (13)	0.0279 (3)	
H7A	0.2623	0.5885	0.8223	0.042*	
H7B	0.4949	0.4707	0.8213	0.042*	
H7C	0.2558	0.5056	0.7380	0.042*	
C8	0.6225 (2)	0.70218 (14)	0.74076 (12)	0.0246 (3)	
H8A	0.5235	0.7468	0.7888	0.037*	
H8B	0.6887	0.7674	0.6828	0.037*	
H8C	0.7578	0.6298	0.7878	0.037*	
C9	0.6047 (2)	0.88745 (13)	0.34042 (12)	0.0218 (3)	
H9A	0.7842	0.8612	0.3361	0.026*	
H9B	0.5595	0.9811	0.3404	0.026*	
C10	0.4806 (2)	0.88266 (13)	0.23604 (12)	0.0232 (3)	
H10	0.5208	0.9356	0.1648	0.028*	
C11	0.3217 (2)	0.81262 (13)	0.23315 (12)	0.0223 (3)	
H11	0.2803	0.7602	0.3043	0.027*	
C12	0.2022 (2)	0.80775 (13)	0.13049 (12)	0.0218 (3)	
C13	0.0192 (2)	0.74014 (14)	0.14118 (13)	0.0257 (3)	
H13	−0.0262	0.6972	0.2147	0.031*	
C14	−0.0978 (3)	0.73432 (15)	0.04654 (14)	0.0303 (4)	
H14	−0.2210	0.6869	0.0557	0.036*	
C15	−0.0356 (3)	0.79759 (15)	−0.06144 (14)	0.0294 (3)	
H15	−0.1168	0.7944	−0.1265	0.035*	
C16	0.1453 (3)	0.86525 (14)	−0.07378 (13)	0.0275 (3)	
H16	0.1881	0.9093	−0.1476	0.033*	
C17	0.2645 (3)	0.86914 (14)	0.02109 (13)	0.0256 (3)	
H17	0.3910	0.9144	0.0116	0.031*	

### Atomic displacement parameters (Å^2^)

**Table:**

	*U*^11^	*U*^22^	*U*^33^	*U*^12^	*U*^13^	*U*^23^
O1	0.0254 (5)	0.0248 (5)	0.0264 (6)	0.0015 (4)	0.0035 (4)	−0.0083 (4)
O2	0.0268 (5)	0.0248 (5)	0.0275 (6)	0.0032 (4)	0.0059 (4)	−0.0055 (4)
C1	0.0183 (6)	0.0232 (6)	0.0187 (7)	−0.0033 (5)	0.0006 (5)	−0.0100 (6)
C2	0.0290 (7)	0.0201 (6)	0.0191 (7)	−0.0029 (6)	0.0011 (5)	−0.0065 (5)
C3	0.0201 (6)	0.0218 (6)	0.0188 (7)	−0.0050 (5)	0.0027 (5)	−0.0059 (5)
C4	0.0189 (6)	0.0276 (7)	0.0210 (8)	−0.0039 (6)	0.0022 (5)	−0.0067 (6)
C5	0.0190 (6)	0.0216 (6)	0.0197 (7)	−0.0021 (5)	−0.0008 (5)	−0.0076 (5)
C6	0.0208 (6)	0.0225 (6)	0.0179 (7)	−0.0045 (5)	0.0004 (5)	−0.0075 (5)
C7	0.0292 (7)	0.0281 (7)	0.0236 (8)	−0.0078 (6)	0.0044 (6)	−0.0047 (6)
C8	0.0226 (7)	0.0296 (7)	0.0226 (8)	−0.0047 (6)	0.0006 (5)	−0.0116 (6)
C9	0.0215 (6)	0.0202 (6)	0.0220 (8)	−0.0041 (5)	0.0030 (5)	−0.0056 (5)
C10	0.0249 (7)	0.0237 (7)	0.0180 (7)	−0.0047 (6)	0.0038 (5)	−0.0040 (5)
C11	0.0228 (6)	0.0225 (6)	0.0174 (7)	−0.0026 (5)	0.0028 (5)	−0.0036 (5)
C12	0.0214 (6)	0.0204 (6)	0.0204 (7)	−0.0008 (5)	0.0016 (5)	−0.0061 (5)
C13	0.0242 (7)	0.0239 (7)	0.0247 (8)	−0.0046 (6)	0.0017 (6)	−0.0035 (6)
C14	0.0257 (7)	0.0306 (7)	0.0354 (9)	−0.0109 (6)	0.0003 (6)	−0.0093 (7)
C15	0.0286 (7)	0.0323 (8)	0.0288 (9)	−0.0058 (6)	−0.0025 (6)	−0.0134 (7)
C16	0.0319 (7)	0.0296 (7)	0.0216 (8)	−0.0076 (6)	0.0054 (6)	−0.0098 (6)
C17	0.0253 (7)	0.0289 (7)	0.0250 (8)	−0.0106 (6)	0.0047 (6)	−0.0095 (6)

### Geometric parameters (Å, °)

**Table:**

O1—C1	1.2946 (16)	C8—H8B	0.9800
O1—H1	0.8400	C8—H8C	0.9800
O2—C5	1.2858 (16)	C9—C10	1.506 (2)
O2—H2	0.8400	C9—H9A	0.9900
C1—C6	1.3941 (18)	C9—H9B	0.9900
C1—C2	1.5026 (19)	C10—C11	1.3212 (19)
C2—C3	1.5296 (19)	C10—H10	0.9500
C2—H2A	0.9900	C11—C12	1.475 (2)
C2—H2B	0.9900	C11—H11	0.9500
C3—C8	1.5260 (19)	C12—C13	1.3922 (19)
C3—C7	1.5276 (19)	C12—C17	1.396 (2)
C3—C4	1.5339 (17)	C13—C14	1.385 (2)
C4—C5	1.5020 (19)	C13—H13	0.9500
C4—H4A	0.9900	C14—C15	1.386 (2)
C4—H4B	0.9900	C14—H14	0.9500
C5—C6	1.4002 (18)	C15—C16	1.381 (2)
C6—C9	1.5005 (19)	C15—H15	0.9500
C7—H7A	0.9800	C16—C17	1.384 (2)
C7—H7B	0.9800	C16—H16	0.9500
C7—H7C	0.9800	C17—H17	0.9500
C8—H8A	0.9800		
			
C1—O1—H1	109.5	C3—C8—H8B	109.5
C5—O2—H2	109.5	H8A—C8—H8B	109.5
O1—C1—C6	119.59 (12)	C3—C8—H8C	109.5
O1—C1—C2	118.58 (11)	H8A—C8—H8C	109.5
C6—C1—C2	121.80 (11)	H8B—C8—H8C	109.5
C1—C2—C3	114.71 (11)	C6—C9—C10	114.36 (11)
C1—C2—H2A	108.6	C6—C9—H9A	108.7
C3—C2—H2A	108.6	C10—C9—H9A	108.7
C1—C2—H2B	108.6	C6—C9—H9B	108.7
C3—C2—H2B	108.6	C10—C9—H9B	108.7
H2A—C2—H2B	107.6	H9A—C9—H9B	107.6
C8—C3—C7	108.85 (12)	C11—C10—C9	126.85 (13)
C8—C3—C2	110.02 (11)	C11—C10—H10	116.6
C7—C3—C2	109.82 (11)	C9—C10—H10	116.6
C8—C3—C4	110.21 (11)	C10—C11—C12	126.52 (13)
C7—C3—C4	109.84 (11)	C10—C11—H11	116.7
C2—C3—C4	108.10 (11)	C12—C11—H11	116.7
C5—C4—C3	114.10 (11)	C13—C12—C17	117.74 (13)
C5—C4—H4A	108.7	C13—C12—C11	119.70 (13)
C3—C4—H4A	108.7	C17—C12—C11	122.56 (12)
C5—C4—H4B	108.7	C14—C13—C12	121.20 (14)
C3—C4—H4B	108.7	C14—C13—H13	119.4
H4A—C4—H4B	107.6	C12—C13—H13	119.4
O2—C5—C6	119.25 (12)	C13—C14—C15	120.14 (14)
O2—C5—C4	118.89 (11)	C13—C14—H14	119.9
C6—C5—C4	121.80 (11)	C15—C14—H14	119.9
C1—C6—C5	119.10 (12)	C16—C15—C14	119.51 (14)
C1—C6—C9	120.71 (12)	C16—C15—H15	120.2
C5—C6—C9	120.07 (11)	C14—C15—H15	120.2
C3—C7—H7A	109.5	C15—C16—C17	120.19 (14)
C3—C7—H7B	109.5	C15—C16—H16	119.9
H7A—C7—H7B	109.5	C17—C16—H16	119.9
C3—C7—H7C	109.5	C16—C17—C12	121.20 (13)
H7A—C7—H7C	109.5	C16—C17—H17	119.4
H7B—C7—H7C	109.5	C12—C17—H17	119.4
C3—C8—H8A	109.5		
			
O1—C1—C2—C3	157.55 (12)	O2—C5—C6—C9	−1.2 (2)
C6—C1—C2—C3	−24.44 (18)	C4—C5—C6—C9	176.16 (12)
C1—C2—C3—C8	−72.89 (14)	C1—C6—C9—C10	90.63 (15)
C1—C2—C3—C7	167.32 (11)	C5—C6—C9—C10	−85.41 (15)
C1—C2—C3—C4	47.50 (15)	C6—C9—C10—C11	2.01 (19)
C8—C3—C4—C5	71.88 (15)	C9—C10—C11—C12	−179.55 (12)
C7—C3—C4—C5	−168.20 (12)	C10—C11—C12—C13	−173.29 (13)
C2—C3—C4—C5	−48.39 (15)	C10—C11—C12—C17	6.5 (2)
C3—C4—C5—O2	−156.07 (12)	C17—C12—C13—C14	−0.2 (2)
C3—C4—C5—C6	26.60 (18)	C11—C12—C13—C14	179.59 (12)
O1—C1—C6—C5	176.74 (12)	C12—C13—C14—C15	−0.7 (2)
C2—C1—C6—C5	−1.3 (2)	C13—C14—C15—C16	0.6 (2)
O1—C1—C6—C9	0.7 (2)	C14—C15—C16—C17	0.4 (2)
C2—C1—C6—C9	−177.34 (12)	C15—C16—C17—C12	−1.3 (2)
O2—C5—C6—C1	−177.27 (12)	C13—C12—C17—C16	1.2 (2)
C4—C5—C6—C1	0.1 (2)	C11—C12—C17—C16	−178.59 (12)

### Hydrogen-bond geometry (Å, °)

**Table:**

*D*—H···*A*	*D*—H	H···*A*	*D*···*A*	*D*—H···*A*
O1—H1···O1^i^	0.84	1.76	2.582 (2)	166
O2—H2···O2^ii^	0.84	1.74	2.569 (2)	167

Symmetry codes: (i) −*x*+2, −*y*+1, −*z*+1; (ii) −*x*, −*y*+2, −*z*+1.
